# Tetrahydrocurcumin protects against sepsis-induced acute kidney injury via the SIRT1 pathway

**DOI:** 10.1080/0886022X.2021.1942915

**Published:** 2021-06-30

**Authors:** Lu Li, Xiaoxi Liu, Shasha Li, Qingyan Wang, Hongru Wang, Menglu Xu, Yanxin An

**Affiliations:** a Department of Nephrology, The First Affiliated Hospital, Xi’an Medical University, Xi’an, China; b Department of General Surgery, The First Affiliated Hospital, Xi’an Medical University, Xi’an, China

**Keywords:** Sepsis, acute kidney injury, tetrahydrocurcumin, SIRT1, inflammation, oxidative stress

## Abstract

Sepsis-induced acute kidney injury (AKI) continues to be associated with poor outcomes in critical care patients. Previous research has revealed that tetrahydrocurcumin (THC) exerts renoprotective effects in multiple nephritic disorders by modulating inflammation and oxidative stress. However, the effects of THC on sepsis-induced AKI and the underlying mechanisms remain unclear. In this study, a mouse model of sepsis-induced AKI, generated by cecal ligation and puncture operation, was used to investigate the protective effects of THC and the role of SIRT1. Histological manifestation and TUNEL analysis were observed to determine the severity of kidney damage. Levels of BUN, SCr, KIM-1, and UAlb/Cr were calculated to assess the renal function. Expressions of IL-1β, IL-6, and TNF-α were measured to evaluate the inflammatory response. MDA content, SOD, GSH, CAT, and GPx activities and DHE staining were analyzed to estimate the degree of oxidative stress. Protein expressions of SIRT1, Ac-p65, and Ac-foxo1 were detected to explore the underlying mechanisms. We observed that THC not only increased the survival rate, improved the kidney function and ameliorated the renal histological damage of septic mice, but also inhibited inflammatory response, prohibited oxidative stress, and prevented cell apoptosis in renal tissues in septic mice. Mechanistically, THC remarkably increased the expression of SIRT1, accompanied by decreased expressions of downstream molecules Ac-p65 and Ac-foxo1. Meanwhile, the beneficial effects of THC were clearly abolished by the SIRT1-specific inhibitor EX527. These results delineate that THC prevents sepsis-induced AKI by suppressing inflammation and oxidative stress through activating the SIRT1 signaling.

**Abbreviation:** Ac-p65: acetylated p65; Ac-foxo 1: acetylated forkhead box O1; AKI: acute kidney injury; BUN: blood urea nitrogen; CAT: catalase; DHE: dihydroethidium; GPx: glutathione peroxidase; GSH: reduced glutathione; IL-1β: Interleukin-1 beta; IL-6: Interleukin-6; KIM-1: kidney injury molecule 1; MDA: malondialdehyde; SCr: serum creatinine; SIRT1: silent information regulator 1; SOD: superoxide dismutase; THC: tetrahydrocurcumin; TNF-α: tumor necrosis factor-alpha; TUNEL: TdT-mediated dUTP Nick-End Labeling; UAlb/Cr: urine micro albumin/creatinine.

## 1. Introduction

Globally, septic acute kidney injury (AKI) is a serious threat to human health, with a very high rate of mortality [1,2]. Epidemiological studies have revealed inextricably associations between AKI and sepsis in critical care patients. Sepsis is an important cause of AKI, while AKI is a frequent and serious complication of sepsis [3]. The occurrence of renal dysfunction secondary to a septic insult usually has an extreme adverse impact on outcomes in hospitalized patients [4]. Unfortunately, there are no effective approaches, with the exception of continuous renal replacement therapy (CRRT) or kidney transplantation, that have been applicated in clinical trials for the treatment of sepsis-induced AKI [5].

The pathophysiologic mechanisms underlying sepsis-induced AKI are complex and not completely elucidated. There is accumulating evidence that alternations in levels of pro-inflammatory and anti-inflammatory factors brought on by sepsis result in an inflammatory response and consequently contribute to AKI [6,7]. Additionally, data from both experimental models and clinical research support the key roles of increased oxidative stress and reactive oxygen species (ROS) in the progression of sepsis-induced AKI [8,9]. Thus, searching for novel agents that target alleviating the inflammatory response and oxidative stress may be a potential strategy for this disease treatment.

Tetrahydrocurcumin (THC), a major active metabolite of curcumin in the gastrointestinal tract, exerts greater physiological and pharmacological activities than those of curcumin, such as stronger anti-inflammatory and antioxidative effects [10]. In particular, several studies have demonstrated that THC effectively prevents drug-related renal damage [11–13]. It was also found that THC was able to preserve renal function in the diabetic state [14]. However, little is known concerning the protective effects of THC against sepsis-induced AKI and the underlying mechanisms have not been clarified until now.

Silent information regulator 1 (SIRT1), a nicotinamide adenine dinucleotide (NAD^+^)-dependent deacetylase, regulates multiple cellular biological processes, including stress responses, cellular senescence, longevity, apoptosis, and inflammation [15,16]. Interestingly, SIRT1 has recently been identified as a novel therapeutic target for the prevention and treatment of several renal diseases [17–19]. Simultaneously, THC has been reported to alleviate diabetic cardiomyopathy mainly by attenuating oxidative stress and fibrosis via increasing the expression of SIRT1 [20]. Whereas, whether SIRT1 and SIRT1-related signaling pathways are involved in the curative effects of THC in mitigating sepsis-induced AKI is still unclear.

The aim of the present study was to explicate the protective roles of THC in the setting of sepsis-induced AKI and to investigate whether its positive effects are connected with the activation of SIRT1 signaling.

## 2. Materials and methods

### 2.1. Animals and ethics statement

Eight-week-old male C57BL/6 mice (weight: 20–25 g) were provided by the Experimental Animal Center of Xi’an Medical University. The mice were housed for 1 week for acclimation at room temperature (25 ± 2 °C, humidity 55 ± 5%), with free access to food and water. All animal study procedures were performed in accordance with the Guide for the Care and Use of Laboratory Animals by the National Academy of Sciences and published by the National Institutes of Health (NIH publication no. 86-23, revised 1996). The animal protocols were approved by the committee of The First Affiliated Hospital of Xi’an Medical University.

### 2.2. Reagents

**Figure 1.**
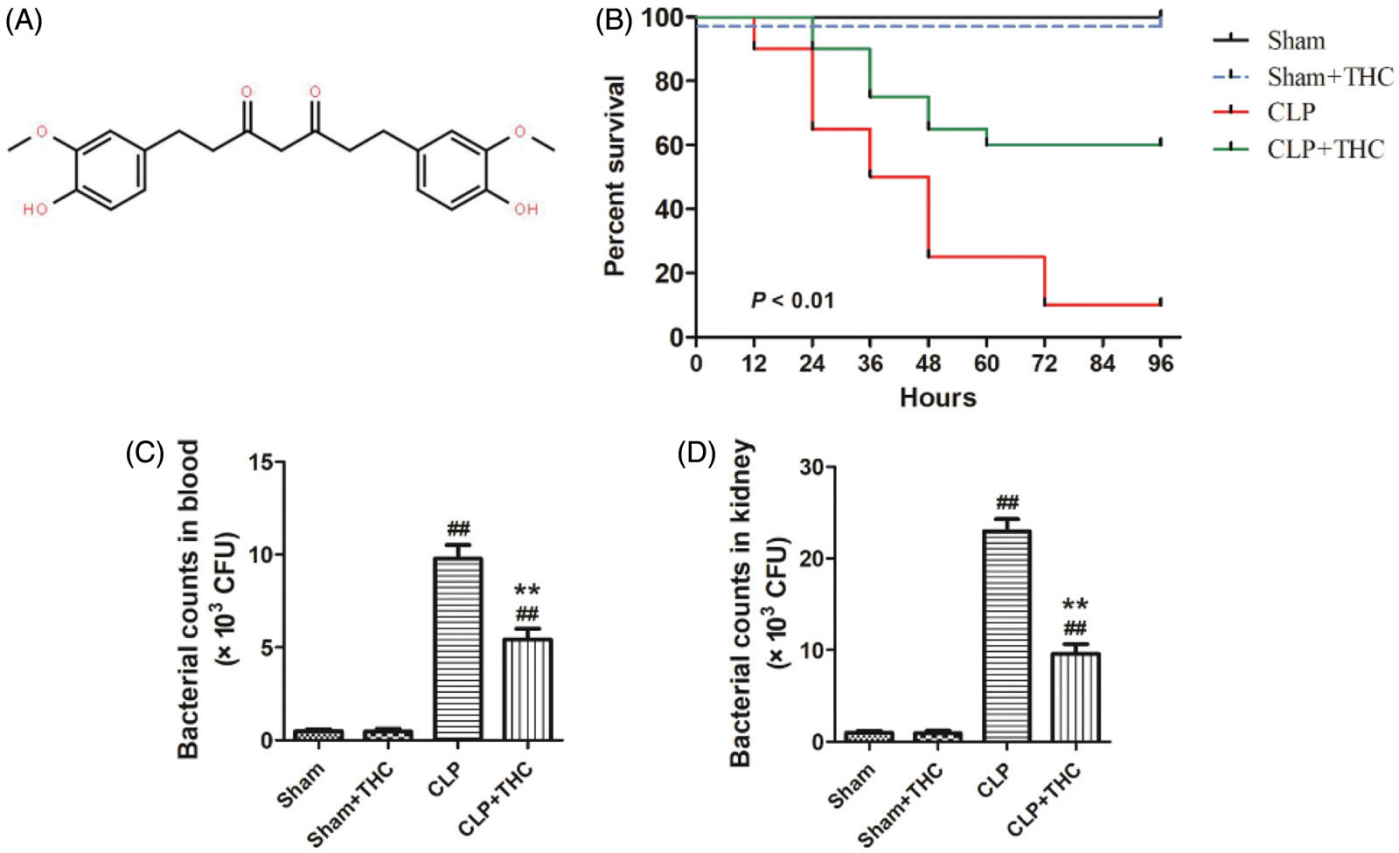
THC improved the survival rate of septic mice and retarded the bacterial growth in blood and kidney after CLP surgery. (A) Molecular formula of THC. (B) Survival curves. (C) Bacterial counts in blood. (D) Bacterial counts in kidney. Data were presented as the mean ± SEM (*n* = 6 in each group). ^##^*p*< .01 vs. the Sham group, **p< .01 vs. the CLP group.

THC (see Figure 1(A) for the molecular formula) and EX527 were purchased from Sigma-Aldrich (St. Louis, MO). Antibodies against SIRT1, TNF-α, IL-1β, and IL-6 were purchased from Abcam (Cambridge, UK). Antibodies against NF-κB, acetylated NF-κB (p65) (Ac-NF-κB), cleaved caspase-3, and β-actin were purchased from Cell Signaling Technology (Boston, MA). Antibodies against forkhead box O1 (foxo1) and acetylated foxo1 (Ac-foxo1) were purchased from Affinity Biosciences (Cincinnati, OH). Rabbit anti-goat, goat anti-rabbit, and goat anti-mouse secondary antibodies were obtained from Zhongshan Company (Beijing, China). Dihydroethidium (DHE) was purchased from Invitrogen (Carlsbad, CA). Kits for assessing superoxide dismutase (SOD), catalase (CAT), reduced glutathione (GSH), glutathione peroxidase (GPx), malondialdehyde (MDA), blood urea nitrogen (BUN), creatinine (Cr), and urine micro albumin (UAlb) levels were purchased from Nanjing Jiancheng Bioengineering Institute (Nanjing, China). The ELISA kit for determining the expression of urinary kidney injury molecule 1 (KIM-1) was obtained from R&D Systems (Minneapolis, MN). Catalog numbers for the main antibodies and kits are shown in Table 1.

**Table 1.** Catalog numbers for the main antibodies and kits.

Name	Catalog number	Company
SIRT1 antibody	ab110304	Abcam (Cambridge, UK)
IL-1β antibody	ab254360	Abcam (Cambridge, UK)
IL-6 antibody	ab208113	Abcam (Cambridge, UK)
TNF-α antibody	ab66579	Abcam (Cambridge, UK)
Ac-NF-κB antibody	#3045	Cell Signaling Technology (Boston, MA)
NF-κB antibody	#8242	Cell Signaling Technology (Boston, MA)
Cleaved caspase-3 antibody	#9661	Cell Signaling Technology (Boston, MA)
β-Actin antibody	#3700	Cell Signaling Technology (Boston, MA)
Ac-foxo1 antibody	AF2305	Affinity Biosciences (Cincinnati, OH)
foxo1 antibody	AF6416	Affinity Biosciences (Cincinnati, OH)
DHE kit	D23107	Invitrogen (Carlsbad, CA)
SOD kit	A001-3-2	Nanjing Jiancheng Bioengineering (Nanjing, China)
MDA kit	A003-1-2	Nanjing Jiancheng Bioengineering (Nanjing, China)
GPx kit	A005-1-2	Nanjing Jiancheng Bioengineering (Nanjing, China)
CAT kit	A007-1-1	Nanjing Jiancheng Bioengineering (Nanjing, China)
BUN kit	C013-2-1	Nanjing Jiancheng Bioengineering (Nanjing, China)
Cr kit	C011-1-1	Nanjing Jiancheng Bioengineering (Nanjing, China)
UAlb kit	E038-1-1	Nanjing Jiancheng Bioengineering (Nanjing, China)
KIM-1 kit	MKM100	R&D Systems (Minneapolis, MN)
SIRT1 activity kit	BML-AK555-0001	Enzo Life Sciences (Farmingdale, NY)

### 2.3. Surgical procedure

Cecal ligation and puncture (CLP) surgery was applied to establish the sepsis model, as described previously [21,22]. All animals were fasted 12 h before the operation and had free access to water. Mice were anesthetized by intraperitoneal injection with pentobarbital sodium (50 mg/kg). A 1 cm incision was made at the midline of the abdomen to expose the cecum. The cecum tightly was ligated tightly with a 6.0 silk suture at its base below the ileo-cecal valve, without causing an intestinal obstruction. After puncturing the cecum twice with a 20-G needle, a small amount of feces was gently squeezed out. Thereafter, the cecum was returned to the peritoneal cavity, and the abdomen was closed with sterile sutures. Fluid resuscitation by subcutaneous injection with pre-warmed normal saline (1 mL/20 g) was necessary. The Sham group was subjected to the same laparotomy procedure used for the CLP group, without ligating and puncturing the cecum.

### 2.4. Experimental protocols

THC and EX527 were first dissolved in ethanol and then diluted in the saline solution. The same volume of vehicle (2% ethanol) or THC (120 mg/kg) was intraperitoneally injected at 30 min and 4 h after CLP. The doses for THC and EX257 were based on previous studies [20,23–25].

Experiment 1 was designed to investigate the influence of THC on sepsis-induced AKI. Mice were randomly assigned to four groups as follows: (1) Sham, (2) Sham + THC, (3) CLP, and (4) CLP + THC. Mice in groups (1) and (2) underwent a sham operation and received vehicle or THC treatment, respectively, for 24 h. Mice in groups (3) and (4) underwent CLP surgery and received vehicle or THC treatment, respectively, for 24 h.

Experiment 2 was designed to probe the molecular mechanisms underlying the effects of THC on sepsis-induced AKI. Mice were randomly assigned to four groups as follows: (i) CLP, (ii) CLP + THC, (iii) CLP + THC + EX527, and (iv) CLP + EX527. Mice in groups (i) and (ii) were treated by the same methods described in experiment 1. Mice in group (iii) were intraperitoneally administered with EX527 (5 mg/kg) at the time of THC injection. Mice in group (iv) were injected with only EX527 (5 mg/kg) at 30 min and 4 h after CLP operation.

Experiment 3 was designed to investigate the effect of THC on the survival rate after CLP. Another 20 mice in each of group (1) and (2) and 10 mice in each of group (3) and (4) were treated by the same methods described in experiment 1. The survival of mice in each group was observed and recorded every 12 h for four days.

### 2.5. Bacterial counts in the kidney

Twenty-four hours after CLP surgery, blood samples were collected in anticoagulation tubes. Kidney tissues were harvested and homogenized in 2 mL of sterile phosphate-buffered saline (PBS) and centrifuged for 5 min. Serial dilutions of the blood and tissue homogenate were evenly spread on nutrient agar plates and incubated at 37 °C for 24 h. Bacterial populations were counted and expressed as colony-forming units (CFU) per organ.

### 2.6. Histomorphology

Paraformaldehyde-fixed and paraffin-embedded mouse kidneys were sliced into 3-μm-thick sections. Hematoxylin–eosin (HE) staining was then performed to observe the morphology of kidney tissues. Five randomly chosen high-power fields in each section were captured by an inverted microscope (Olympus, Tokyo, Japan). The damage quantification from five areas corresponding to the renal tissue was graded using the following parameters: cellular edema, cell apoptosis, necrocytosis, inflammatory cell infiltration, and hemorrhage based on a four-score system (0, histopathological changes ≤10%; 1 = 11–25%; 2 = 26–50%; 3 = 51–75%; and 4 = 76–100%). The mean score for each parameter was calculated and subjected to statistical analysis [26–28].

### 2.7. Measurement of SCr, BUN, UAlb/Cr, and KIM-1

Blood samples were collected to measure BUN and SCr levels using a microplate reader (Thermo Scientific, Waltham, MA). Urine samples were collected to measure the expression levels of KIM-1and UAlb/Cr according to the instructions provided with the respective ELISA kits.

### 2.8. Detection of ROS level

The kidney was rapidly frozen in liquid nitrogen and sectioned into 3-μm-thick slices. The frozen tissue sections were stained with a DHE reaction mixture in a dark humidified chamber. Each sample was examined using an Olympus FV1000 laser confocal microscope (Tokyo, Japan). The fluorescence intensity, determined using ImageJ software (NIH, Bethesda, MD), was detected to evaluate ROS generation in kidney tissues.

### 2.9. Determination of MDA content, and SOD, GSH, CAT, and GPx activities

Commercial kits for determining the content of MDA and activities of SOD, GSH, CAT, and GPx in kidney tissues were used according to the manufacturers’ instructions. The results were analyzed spectrophotometrically using a SpectraMax M5 device (Molecular Devices, San Jose, CA).

### 2.10. Quantitative reverse transcription polymerase chain reaction (qRT-PCR)

Total RNA was extracted from kidney tissues using TRIzol reagent. Thereafter, cDNA was synthesized using the High Capacity cDNA Reverse Transcription Kit (Invitrogen, Carlsbad, CA) following the manufacturer’s protocol. The CFX96 RT-PCR system was used for qRT-PCR procedures. Relative levels of target genes were determined using the 2^−ΔΔCt^ method. The primer sequences are shown in Table 2.

**Table 2.** Sequences of primers used for quantitative RT-PCR assays.

Gene	Forward (5′–3′)	Reverse (5′–3′)
IL-1β	TGCCACCTTTTGACAGTGATGAG	TGATGTGCTGCTGCGAGATTT
IL-6	AGGATACCACTCCCAACAGACC	GCACAACTCTTTTCTCATTTCCAC
TNF-α	ACTCCAGGCGGTGCCTATG	GTGAGGGTCTGGGCCATAGAA
Bax	CACAGCGTGGTGGTACCTTA	TCTTCTGTACGGCGGTCTCT
Bcl2	TCGCAGAGATGTCCAGTCAG	ATGCCGGTTCAGGTACTCAG
GAPDH	AGAACATCATCCCTGCATCC	AGTTGCTGTTGAAGTCGC

### 2.11. Measurement of SIRT1 deacetylase activity

SIRT1 deacetylase activity was measured by a fluorometric assay according to the manufacturer’s instructions. After Developer II was added to each well, the reaction mixture was incubated at 37 °C for 1 h. Fluorescence was read using a SpectraMax M5 instrument with an excitation wavelength of 360 nm and emission wavelength of 460 nm.

### 2.12. Western blotting

Total protein was extracted from kidney tissues and quantified using a bicinchoninic acid (BCA) Kit (Solarbio, Beijing, China). Samples containing 30 μg of proteins were separated by SDS-PAGE and then transferred to polyvinylidene difluoride membranes (Millipore, Billerica, MA). After blocking with 5% nonfat milk for 2 h at room temperature, the membranes were incubated overnight at 4 °C with primary antibodies specific for SIRT1 (1:1000), TNF-α (1:1000), IL-1β (1:1000), IL-6 (1:1000), Ac-NF-κB (1:500), NF-κB (1:500), Ac-foxo1 (1:500), foxo1 (1:500), cleaved caspase-3 (1:500), and β-actin (1:5000). Next, the membranes were incubated with appropriate horseradish peroxidase (HRP)-conjugated secondary antibodies, and protein bands were analyzed using Image Lab 5.2.1 (Bio-Rad Laboratories, Hercules, CA).

### 2.13. Statistical analysis

Data were analyzed using GraphPad Prism 5.0 (San Diego, CA) and results were expressed as means ± standard error of the mean (SEM). One-way ANOVA was adopted to assess differences among groups, followed by post hoc *t*-tests with Bonferroni’s correction. The Kaplan–Meier method and log-rank test were used for the survival analysis. A value of *p* less than .05 was considered statistically significant.

## 3. Results

### 3.1. THC improved the survival rate of septic mice and retarded the bacterial growth in the blood and kidney after CLP surgery

As shown in Figure 1(B), after four days of observation, the survival rates in both the Sham and Sham + THC groups were 100%. As for the septic mice that undergone CLP surgery, THC administration significantly increased the survival rate from 10% in the CLP group to 60% in the CLP + THC group, which indicated that THC showed an outstanding protective effect in reducing mortality in septic mice.

Meanwhile, 24 hours after CLP operation, bacterial counts were significantly raised in the blood and renal tissue of the CLP group than that in the Sham group. Whereas, THC administration markedly inhibited bacterial growth in the blood and kidney of the CLP + THC group, which suggested that THC exhibited a strong anti-bacterial capability in septic mice (Figure 1(C,D)).

### 3.2. THC improved kidney function and ameliorated renal histological damage in septic mice

**Figure 2.**
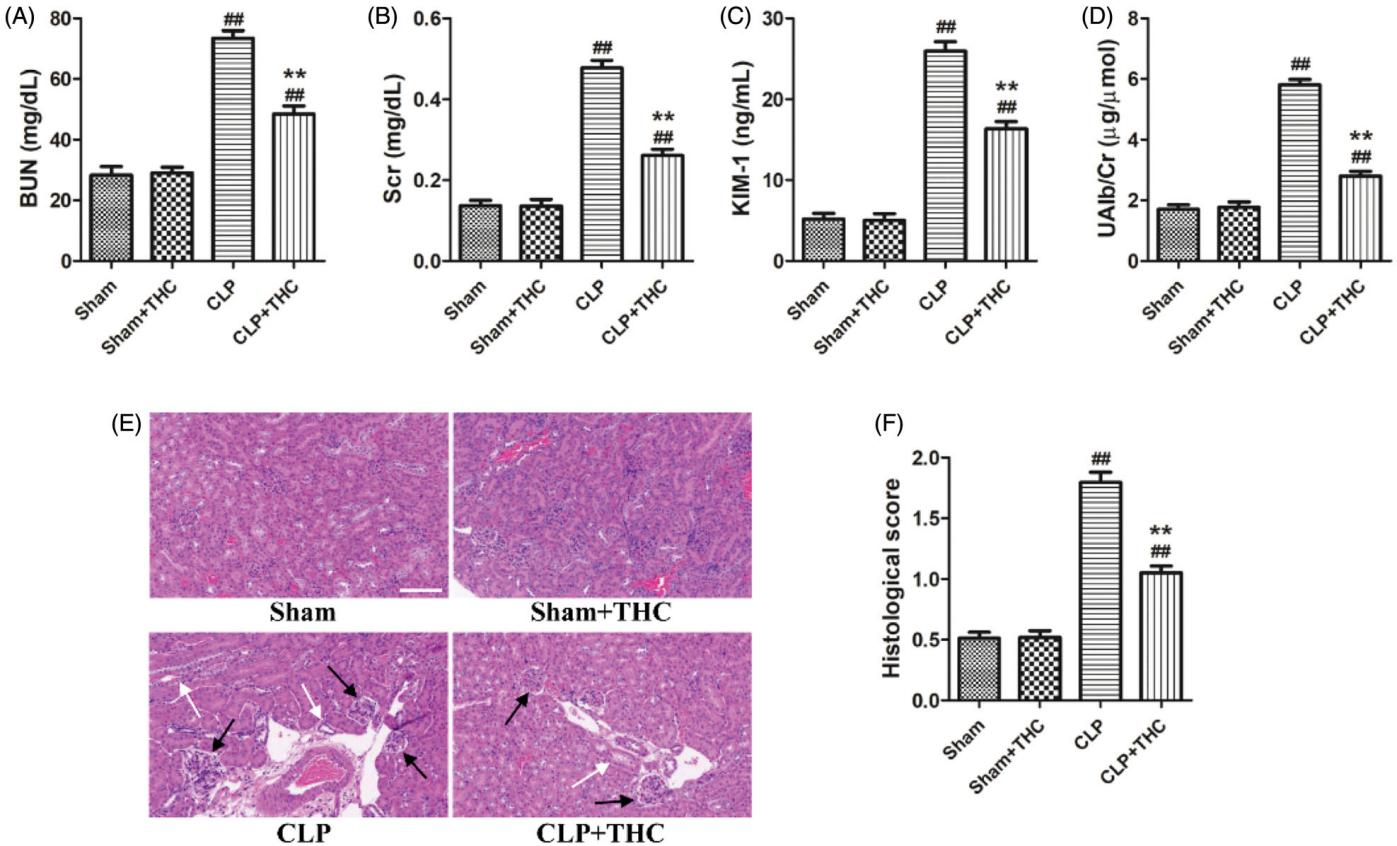
THC improved the kidney function and ameliorated renal histological damage in septic mice. (A) The serum level of BUN. (B) The serum level of Cr. (C) The urine level of KIM-1. (D) The urine level of UAlb/Cr. (E) Representative images of HE staining of renal histological changes (black arrow: glomerulus degeneration and necrosis, white arrow: renal tubules edema) (magnification ×400, scale bar = 50 μm). (F) Histological score. Data were presented as the mean ± SEM (*n* = 6 in each group). ^##^*p*< .01 vs. the Sham group, **p< .01 vs. the CLP group.

The levels of several biomarkers including BUN, SCr, KIM-1, and UAlb/Cr were detected to identify the role of THC on kidney function in septic mice. As shown in Figure 2(A–D), the levels of these parameters were markedly elevated in the CLP group than those in the Sham group, while THC treatment significantly mitigated these damages. Similarly, histological changes were consistent with the results for kidney function in each group (Figure 2(E,F)). Obvious renal tissue changes, such as glomerular necrosis and hypertrophy, as well as cast formation in the tubules were observed in the CLP group, which had a higher histological score of kidney injury than that in the Sham group. In contrast, the septic mice treated with THC had a markedly reduced histological score of kidney injury in the CLP + THC group.

### 3.3. THC promoted the expression of SIRT1 in renal tissue in septic mice

**Figure 3.**
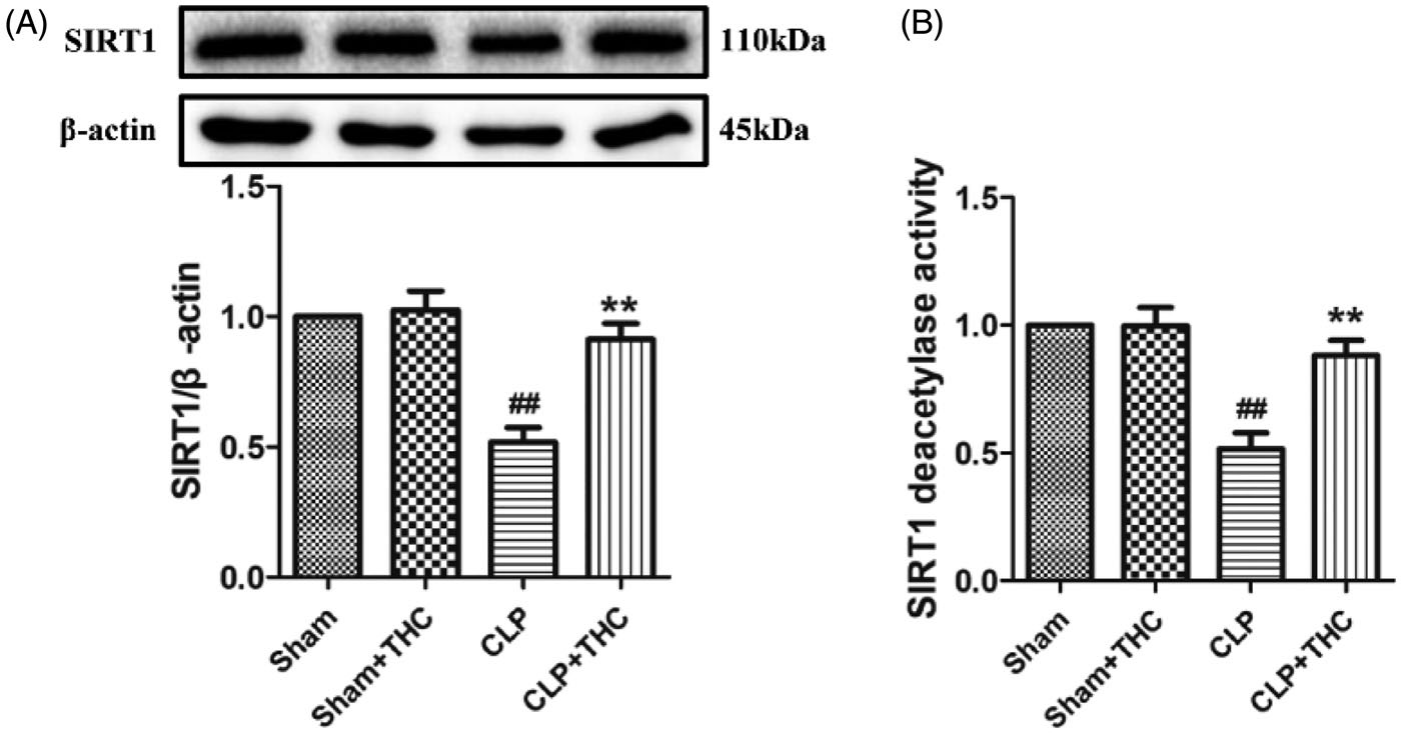
THC promoted the expression of SIRT1 in renal tissues in septic mice. (A) SIRT1 expression. (B) Relative SIRT1 activity. Data were presented as the mean ± SEM (*n* = 6 in each group). ^##^*p*< .01 vs. the Sham group, **p< .01 vs. the CLP group.

Intriguingly, compared with the Sham group, the expression and deacetylation activity of SIRT1 were significantly decreased in the renal tissue of the CLP group. While the administration of THC brought about a notably up-regulation in the expression and deacetylation activity of SIRT1 in the CLP + THC group (Figure 3), suggesting that SIRT1 signaling might mediate the protective action of THC against AKI in septic mice.

### 3.4. Ex527 blocked the THC-induced attenuation of renal damage and kidney dysfunction in septic mice

**Figure 4.**
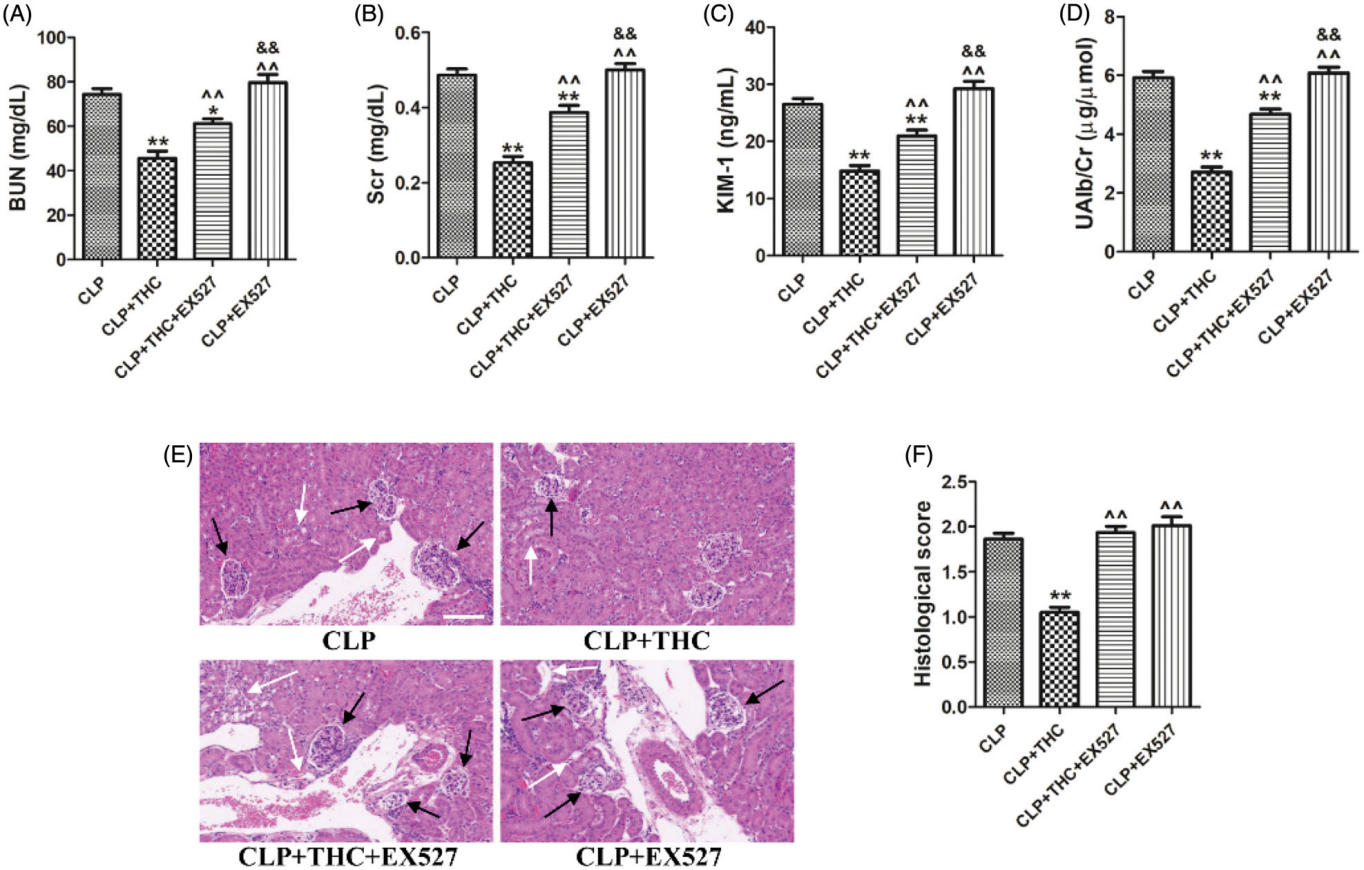
EX527 blocked THC induced attenuation of renal damage and kidney dysfunction in septic mice. (A) The serum level of BUN. (B) The serum level of Cr. (C) The urine level of KIM-1. (D) The urine level of UAlb/Cr. (E) Representative images of HE staining of renal histological changes (black arrow: glomerulus degeneration and necrosis, white arrow: renal tubules edema) (magnification ×400, scale bar = 50 μm). (F) Histological score. Data were presented as the mean ± SEM (*n* = 6 in each group). *^,^**p< .05/.01 vs. the CLP group, ^^^^*p*< .01 vs. the CLP + THC group, ^&&^*p*< .01 vs. the CLP + THC + EX527 group.

To verify whether SIRT1 played a critical role in the underlying molecular mechanisms of THC against CLP-induced renal damage, we further employed the SIRT1-specific antagonist EX527 in *in vivo* experiments. As shown in Figure 4, despite THC improved the kidney function and ameliorated renal histological damage in septic mice, these protective effects of THC were markedly reversed by EX527, indicating that THC attenuated renal damage and kidney dysfunction in septic mice in a SIRT1-dependent manner.

### 3.5. Ex527 blunted the THC-induced amelioration of inflammatory response in renal tissue in septic mice

**Figure 5.**
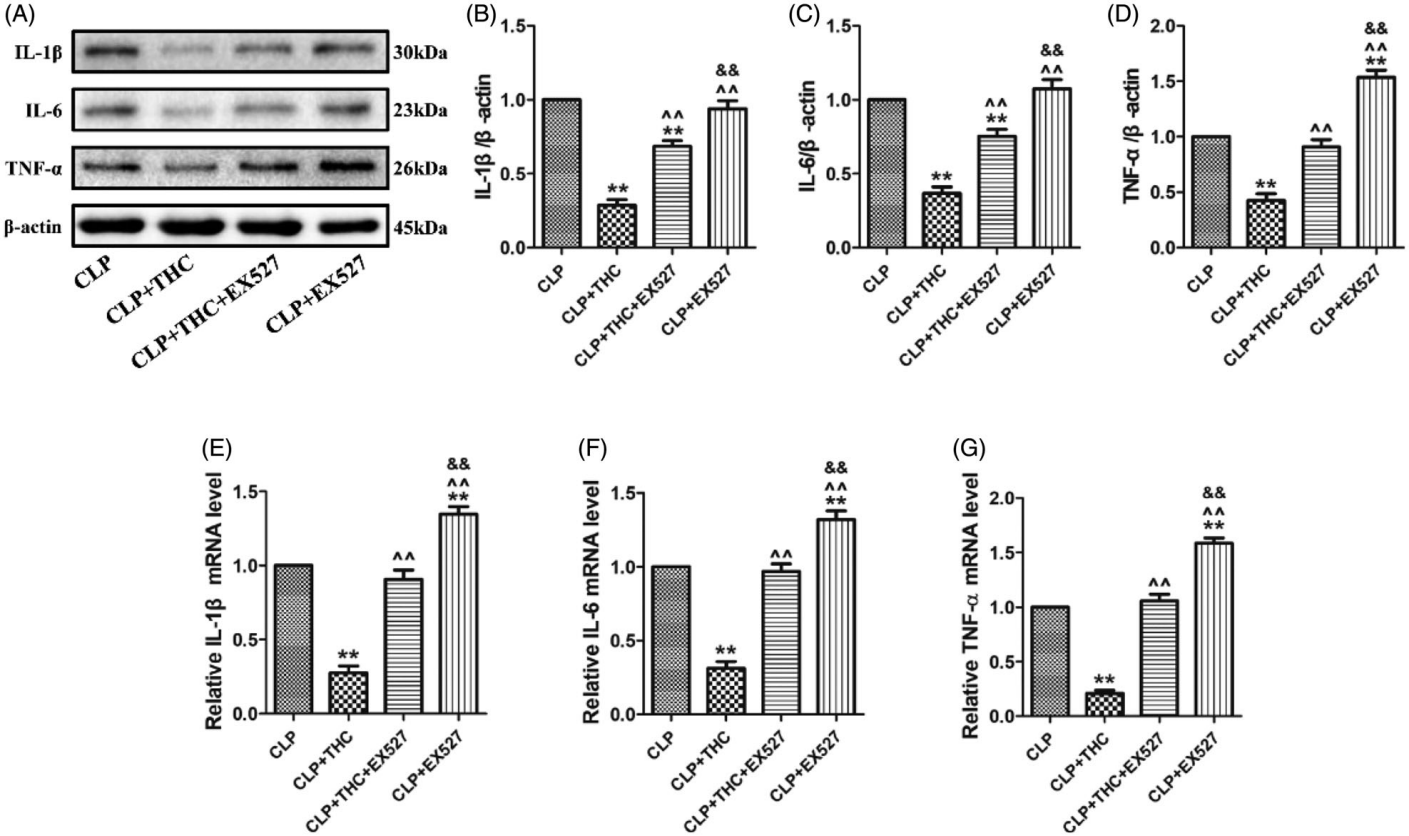
EX527 blunted THC induced amelioration of inflammatory response in renal tissues in septic mice. (A) Representative blots. (B) IL-1β expression. (C) IL-6 expression. (D) TNF-α expression. (E) Relative IL-1β mRNA level. (F) Relative IL-6 mRNA level. (G) Relative TNF-α mRNA level. Data were presented as the mean ± SEM (*n* = 6 in each group). ^**^p< .01 vs. the CLP group, ^^^^*p*< .01 vs. the CLP + THC group, ^&&^*p*< .01 vs. the CLP + THC + EX527 group.

Increases in inflammatory chemokines and cytokines occur during the progression of sepsis-derived AKI. To investigate the anti-inflammatory effects of THC, we examined the expressions of several pro-inflammatory cytokines in renal tissue at the protein and mRNA levels. As shown in Figure 5, the protein and mRNA expressions of IL-1β, IL-6, and TNF-α were all markedly elevated in renal tissue of mice with CLP-induced sepsis, which were consistent with findings from other studies [26,29]. Importantly, these enhanced inflammatory responses were notably suppressed by THC administration in the CLP + THC group. However, the above anti-inflammatory actions of THC were markedly abolished by EX527, which suggested that SIRT1 signaling played a pivotal role in the amelioration effects of THC on inflammatory response in renal tissue in septic mice.

### 3.6. Ex527 counteracted the THC-induced inhibition of oxidative stress in renal tissue in septic mice

**Figure 6.**
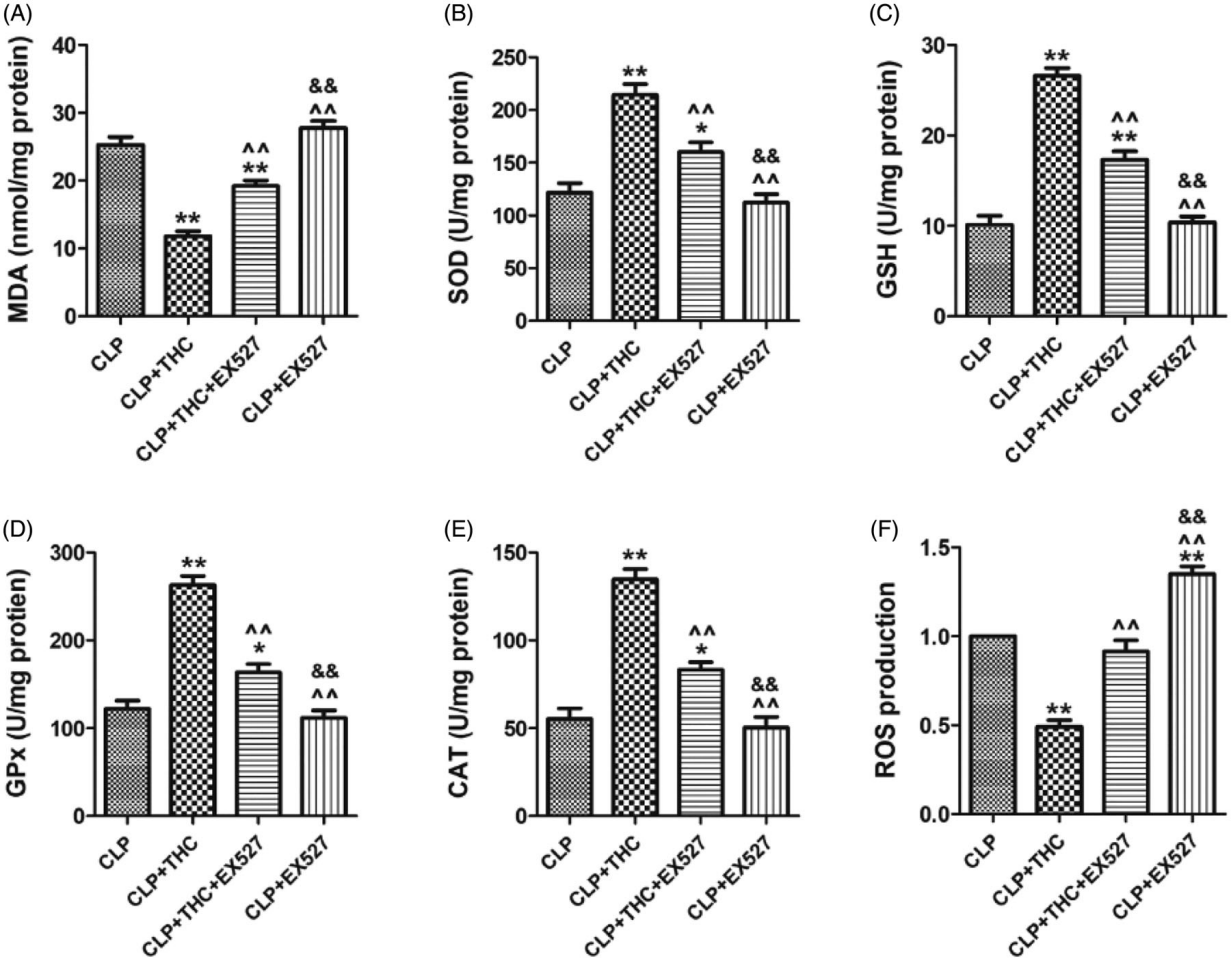
EX527 counteracted THC induced inhibition of oxidative stress in renal tissues in septic mice. (A) Renal MDA content. (B) Renal SOD activity. (C) Renal GSH activity. (D) Renal GPx activity. (E) Renal CAT activity. (F) Renal ROS production. Data were presented as the mean ± SEM (*n* = 6 in each group). ^*,**^p< .05/.01 vs. the CLP group, ^^^^*p*< .01 vs. the CLP + THC group, ^&&^*p*< .01 vs. the CLP + THC + EX527 group.

As shown in Figure 6(A–E), THC significantly increased the enzymatic activities of key endogenous antioxidants (i.e., SOD, GSH, CAT, and GPx) in renal tissue in the CLP + THC group. In the meantime, the content of MDA, an endogenous genotoxic product, was also markedly decreased. However, all these phenomena were dramatically abolished by EX527. Besides, compared with the CLP group, THC also exhibited its antioxidant capability by inhibiting ROS generation in the renal tissue of septic mice. While EX527 administration prominently counteracted this curative effect as well (Figure 6(F)). These data indicated that SIRT1 signaling participated in the ameliorative effects of THC against renal oxidative stress in septic mice.

### 3.7. EX527 abolished the THC-induced prevention of cell apoptosis in renal tissue in septic mice

**Figure 7.**
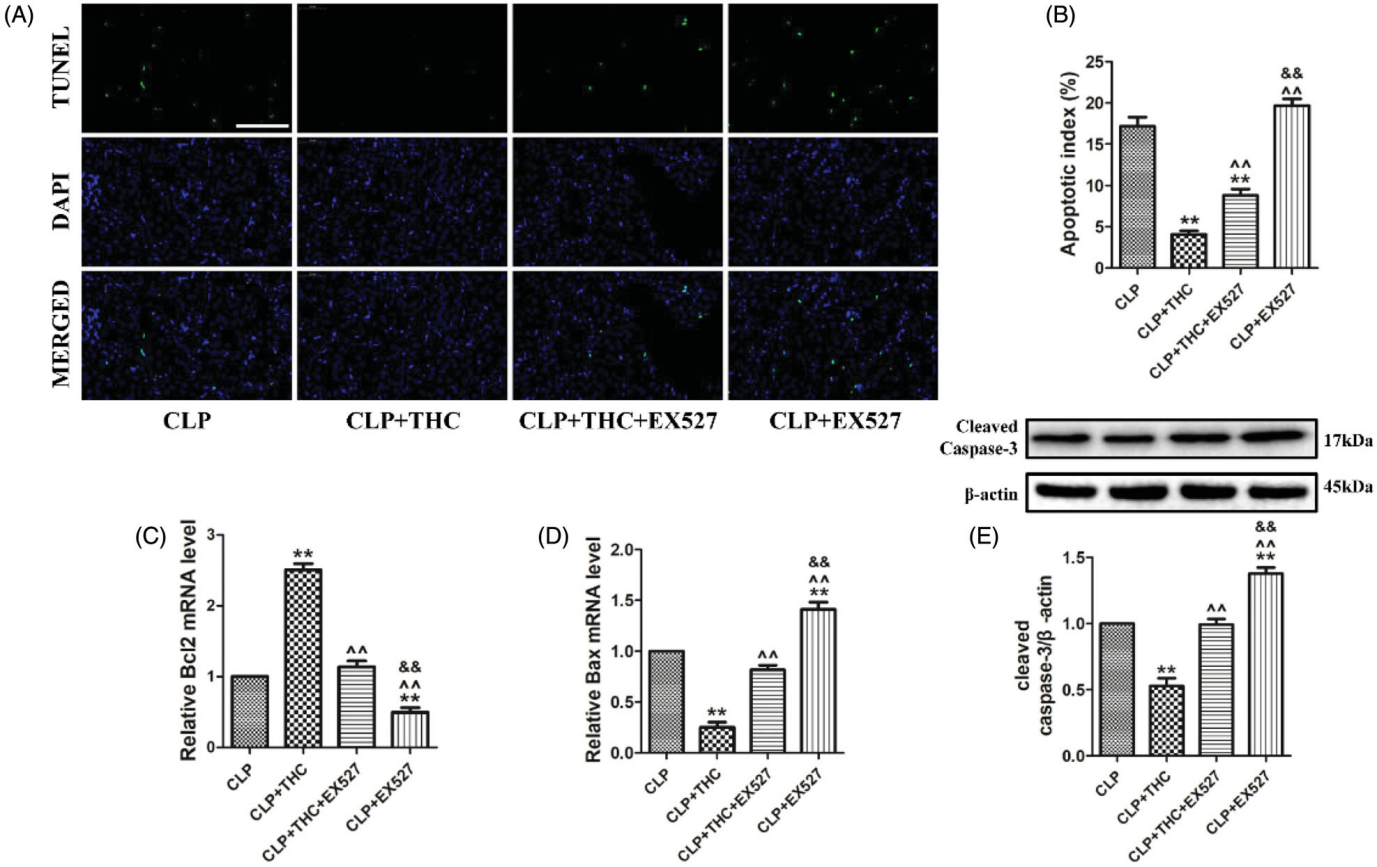
EX527 abolished THC induced prevention of cell apoptosis in renal tissue in septic mice. (A) Representative images of TUNEL staining (scale bar = 50 μm). The apoptotic cells were detected by TUNEL (green) and the nuclei were detected by DAPI (blue). (B) Cellular apoptotic index. (C) Relative Bcl2 mRNA level. (D) Relative Bax mRNA level. (E) Cleaved caspase-3 expression. Data were presented as the mean ± SEM (*n* = 6 in each group). **p< .01 vs. the CLP group, ^^^^*p*< .01 vs. the CLP + THC group, and ^&&^*p*< .01 vs. the CLP + THC + EX527 group.

To investigate the effect of THC on CLP-induced apoptosis in septic mice, TUNEL staining was used to evaluate septic AKI at the cellular level. As shown in Figure 7(A,B), compared with the CLP group, THC treatment significantly prevented cell apoptosis in renal tissue in septic mice, as evidenced by a remarkable decreased number of TUNEL-positive nuclei and the apoptotic ratio. However, EX527 abolished the anti-apoptotic effect of THC against septic AKI following CLP operation. Additionally, pro-apoptotic markers (Bax and cleaved caspase-3) and anti-apoptotic marker (Bcl2) were detected. In accordance with the TUNEL staining results, THC greatly reduced Bax and cleaved caspase-3 expressions as well as elevating Bcl2 expression in the renal tissue of septic mice, while the THC-induced suppression of the expressions of apoptosis-related proteins was significantly invalidated by EX527 (Figure 7(C–E)). Collectively, these data demonstrated that THC displayed a protective property against renal apoptosis in septic mice via SIRT1 signaling.

### 3.8. THC protected renal tissue from sepsis-induced AKI via the activation of SIRT1

**Figure 8.**
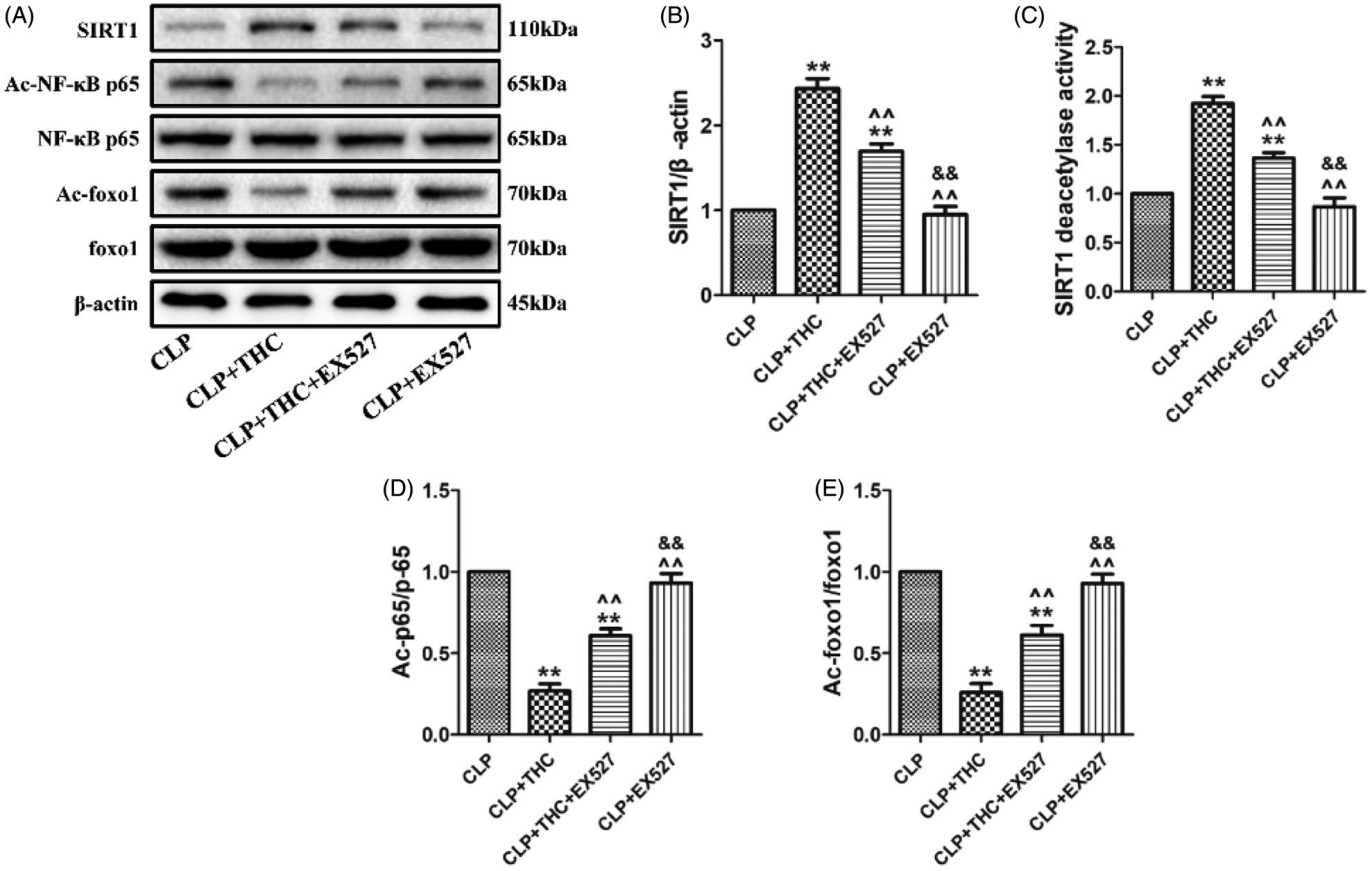
THC could protect renal tissue from sepsis-induced AKI through the activation of SIRT1. (A) Representative blots. (B) SIRT1 expression. (C) Relative SIRT1 activity. (D) Ac-NF-κB expression. (E) Ac-foxo1 expression. Data were presented as the mean ± SEM (*n* = 6 in each group). ^**^p< .01 vs. the CLP group, ^^^^*p*< .01 vs. the CLP + THC group, ^&&^*p*< .01 vs. the CLP + THC + EX527 group.

As displayed in Figure 8(A–C), although THC brought about a notably up-regulation in the expression and deacetylation activity of SIRT1 in the CLP + THC group, EX527 administration obviously blunted this effect. Meanwhile, as important downstream deacetylation targets of SIRT1, the acetylation levels of NF-κB and foxo1 were dramatically decreased in the presence of EX527, which further indicated the decline in the deacetylation capacity of SIRT1 in the CLP + THC group (Figure 8(D,E)). Of note, the use of EX527 abolish all the positive effects of THC in the renal tissue of septic mice in our above *in vivo* experiments. In summary, these data demonstrated that THC could protect the renal tissue from sepsis-induced AKI via the activation of SIRT1 signaling.

## 4. Discussion

The current research showed that in the setting of sepsis-induced AKI, THC could protect renal function and attenuate AKI by suppressing oxidative stress and inflammation, which might be mediated by activating the SIRT1 signaling pathway. For the first time, this study confirmed the renoprotective effects and provided insight into the underlying mechanisms of THC against septic AKI.

Worldwide, the prevalence of AKI in patients admitted to the hospital or intensive care unit (ICU) is astonishing [30]. AKI is a complex clinical syndrome defined by a rapid increase in serum Cr level and a sudden loss of the kidney’s excretory function, which has been seen as a major public health issue associated with raised morbidity and mortality [1,2]. Despite technological advances in renal replacement therapies, the majority of patients remain to be connected with poor outcomes and grievous renal impairment [5]. Significantly, sepsis is reported to be a crucial trigger for AKI in critically ill patients. Comparative studies have revealed that patients with sepsis-induced AKI carried a more pronounced variation in kidney function, a more drastic change in renal histology, and a higher rate of overall mortality [31]. Therefore, effective therapeutic strategies are imperative to be developed to relieve the symptoms and improve the prognosis of this severe clinical entity.

THC is the principal metabolite of curcumin found in the gastrointestinal tract, which is highly stable in all physiological buffers [10]. Crucially, previous studies have observed the favorable effects of THC in alleviating renal damage. For example, Song et al. noted that THC exhibited protective effects against cisplatin-induced renal damage in rats [11]. Park et al. provided the therapeutic evidence that THC ameliorated tacrolimus-induced nephrotoxicity [12]. Additionally, Pari and Murugan demonstrated that THC protected against the renal damage associated with chloroquine [13]. In the present study, we first found that THC improved the survival rate of septic mice. Since bacterial infection is a major contributor leading to systemic sepsis [32,33], we further detected that THC also retarded the bacterial growth in the blood and kidney in the CLP + THC group. Meanwhile, several critical markers, including BUN, SCr, KIM-1, and UAlb/Cr were used to assess the deterioration of renal function [34,35], which were markedly decreased after THC administration in septic mice. In addition, histopathological alterations such as glomerular necrosis and hypertrophy, and renal tubules edema, were all mitigated by THC as well. Hence, the treatment of THC may be a potential therapy for the intervention of sepsis-induced AKI.

Currently, the mechanisms of sepsis-induced AKI are complex and still under investigation. Evidences from both experimental models and clinical research have suggested that inflammation plays a crucial role in the pathogenesis of sepsis-induced AKI [6,7]. Following the occurrence of systemic sepsis, increases in circulating inflammatory cytokines and leucocyte activity result in a further proinflammatory response in the kidney, thus causing microcirculatory disturbance, endothelial dysfunction, and dysfunctional tubular-glomerular feedback, ultimately leading to renal damage [36]. Therefore, to relive the inflammatory response will be a very promising strategy for treating sepsis-induced AKI. Intriguingly, there is substantial evidence that THC exerts a strong anti-inflammatory activity in multiple organs under different pathological conditions. Pan et al. revealed that THC mitigated acute hypobaric hypoxia-induced cerebral edema and inflammation [37]. Chen et al. reported that THC ameliorated allergic airway inflammation in asthmatic mice [23]. Kakkar et al. demonstrated that the topical delivery of THC lipid nanoparticles effectively inhibited skin inflammation [38]. More importantly, THC could ameliorate cisplatin-induced renal damage by regulating inflammation [11]. In the present study, we found that in the CLP-induced septic mice, THC also markedly suppressed the protein and mRNA expressions of inflammatory chemokines (IL-1β, IL-6, and TNF-α) in renal tissue, indicating that THC could protect against sepsis-induced AKI partly through exhibiting its anti-inflammatory effects.

Oxidative stress, triggered by excessive ROS production, is another cornerstone in the progression of sepsis-induced AKI. Continuously boosted oxidative stress unduly exhausts endogenous antioxidant enzymes, which leads to membranous lipid peroxidation, protein and DNA oxidation, and finally results in the destruction of cell structure and function as well as the damage of renal tissue [8,9]. In our study, ROS generation was obviously increased in the kidneys of septic mice compared with the Con group. In addition, the activity levels of SOD, GSH, CAT, and GPx decreased and the content of MDA increased, which were consistent with the results of previous investigations [27,39]. Numerous studies have discovered that the inhibition of oxidative stress is a vital approach implicated in mitigating sepsis-induced AKI [27,39,40]. Notably, THC has been widely reported to show a stronger antioxidant property than that of curcumin, and it plays a noteworthy protective role in the renal tissue of nephritic model cause by oxidative stress. Lau et al. demonstrated that dietary THC therapy improved expression of antioxidant proteins in the nephrectomized rats [41]. Okada et al. indicated that THC ameliorated oxidative stress-induced renal injury in mice [42]. As expected, our results also showed that THC could attenuate excessive ROS generation and recover the function of antioxidative systems, highlighting its potency in alleviating oxidative damage in the renal tissue of septic mice.

When confronting injuries such as sepsis, ischemia, and toxicants, cell death presents in renal tissue and severely affects the quality of kidney function [43]. Apoptosis, a major type of cell death, is mediated by multiple pathways in response to inflammation and oxidative stress, thus greatly contributing to the consequences of kidney damage and dysfunction [44,45]. Consistently, our study revealed that the apoptotic response in the renal tissue of septic mice is strikingly enhanced, while THC treatment significantly prevented cell apoptosis with a remarkably decreased number of TUNEL-positive nuclei and apoptotic ratio. Furthermore, the anti-apoptotic capacity of THC induced strong downregulation of Bax and cleaved caspase-3 and upregulation of Bcl2. Taken together, these observations supported a role for THC in preventing cell apoptosis in sepsis-induced AKI.

Although the above beneficial effects of THC have been thoroughly described, little is known about the molecular mechanisms involved in these processes. SIRT1, an NAD^+^-dependent protein deacetylase, is regarded as a major modulator of sepsis-induced AKI for its role in mitigating oxidative stress and inflammation [15,16]. The actions of SIRT1 primarily rely on the deacetylation of its downstream targets, like foxo1 and NF-κB [46]. On the one hand, the deacetylation of foxo1 by SIRT1 increases the synthesis of antioxidants and elevates the cellular antioxidative capacity [47]. On the other hand, SIRT1 can deacetylate NF-κB subunit p65 to suppress the expressions of inflammatory cytokines and decrease the cellular inflammatory response [48]. Therefore, decreases in the expression or activity of SIRT1 are closely associated with the increases of oxidative stress and inflammation. Simultaneously, THC has been reported to alleviate diabetic cardiomyopathy mainly by attenuating oxidative stress and fibrosis via activating the expression of SIRT1 [20]. Accordingly, we evaluated the connection between SIRT1 signaling and the protective effects of THC in this disease model. Within expectation, the expression and deacetylation activity of SIRT1 were significantly decreased in renal tissue of the CLP group. While the administration of THC brought about a notably up-regulation in the expression and deacetylation activity of SIRT1 in the CLP + THC group. More importantly, THC induced improvement on kidney function and amelioration of renal histological damage were obviously blocked by EX527. Additionally, the inhibitory effects of THC on inflammatory response, oxidative stress and cell apoptosis in the renal tissue in septic mice were blunted by EX527 as well. Furthermore, the acetylation levels of foxo1 and NF-κB were dramatically decreased in the presence of EX527, which indicated that SIRT1 is the key determinant of deacetylation of foxo1 and NF-κB, subsequently affecting the inflammatory response and oxidative stress. Consequently, these data confirmed that THC could protect the renal tissue from sepsis-induced AKI via the activation of SIRT1 and its related downstream signaling.

In summary, this study demonstrated that in the model of CLP-induced sepsis, THC exerted beneficial effects on sepsis-induced AKI, as verified by increased survival rate, improved kidney function and ameliorated renal histological damage of septic mice. Moreover, the inflammatory response, oxidative stress and cell apoptosis in the renal tissue of septic mice were also prohibited by THC treatment. Mechanistically, the protective capabilities of THC were mediated in part by the SIRT1 signaling axis and related antioxidative and anti-inflammatory activities. These results provide a basis for the use of THC to treat septic AKI. However, further studies are needed to explore the upstream and (or) downstream signaling molecules of SIRT1 to establish a more comprehensive understanding of the mechanisms underlying the protective actions of THC in this disease model.

## Data Availability

The data used to support the findings of this study are available from the corresponding author upon request.
